# Sulfur-regulated control of the *met-2*^*+*^ gene of *Neurospora crassa* encoding cystathionine β-lyase

**DOI:** 10.1186/1756-0500-6-259

**Published:** 2013-07-08

**Authors:** Brad S Reveal, John V Paietta

**Affiliations:** 1 Department of Biochemistry and Molecular Biology, Wright State University, Dayton, OH 45435, USA

**Keywords:** Cystathionine β-lyase, *met-2*^*+*^, Transsulfuration, Sulfur gene regulation, CYS3 regulator, *Neurospora crassa*

## Abstract

**Background:**

Cystathionine β-lyase performs an essential role in the transsulfuration pathway by its primary reaction of forming homocysteine from cystathionine. Understanding how the *Neurospora crassa met-2*^*+*^ gene, which encodes cystathionine β-lyase, is regulated is important in determining the basis of the cellular control of transsulfuration. The aim of this study was to determine the nature of a potential regulatory connection of *met-2*^*+*^ to the *Neurospora* sulfur regulatory network.

**Findings:**

The cystathionine β-lyase (*met-2*^*+*^) gene was cloned by the identification of a cosmid genomic clone capable of transforming a *met-*2 mutant to methionine prototrophy and subsequently characterized. The gene contains a single intron and encodes a protein of 457 amino acids with conserved residues predicted to be important for catalysis and pyridoxal-5′-phosphate co-factor binding. The expression of *met-2*^*+*^ in wild-type *N. crassa* increased 3.1-fold under sulfur-limiting growth conditions as compared to the transcript levels seen under high sulfur growth conditions (i.e., repressing conditions). In a Δ*cys-3* strain, *met-2*^*+*^ transcript levels were substantially reduced under either low- or high-sulfur growth conditions. In addition, the presence of CYS3 activator binding sites on the *met-2*^*+*^ promoter was demonstrated by gel mobility shift assays.

**Conclusions:**

In this report, we demonstrate the sulfur-regulated expression of the *met-2*^+^ gene and confirm its connection to the *N. crassa* sulfur regulatory circuit by the reduced expression observed in a Δ*cys-3* mutant and the *in vitro* detection of CYS3 binding sites in the *met-2*^*+*^ promoter. The data further adds to our understanding of the regulatory dynamics of transsulfuration.

## Findings

### Background

**Figure 1:**
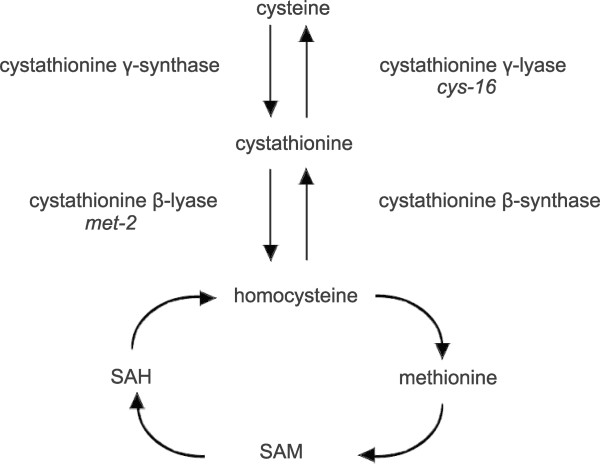
**Pathway of transsulfuration and methionine synthesis in *Neurospora crassa*.** Cystathionine β-lyase, encoded by the *met-2*^*+*^ gene, is shown in its role in the conversion of cystathionine to homocysteine. Cystathionine γ-lyase (encoded by *cys-16*^*+*^), which converts cystathionine to cysteine, has been characterized previously [19]. Abbreviations: SAM, S-adenosylmethionine; SAH, S-adenosylhomocysteine.

Cystathionine β-lyase (E.C. 4.4.1.8; also known as β-cystathionase) catalyzes the conversion of cystathionine to homocysteine, ammonia and pyruvate. Cystathionine β-lyase plays an important role in transsulfuration in that it allows for the utilization of the intracellular pool of cystathionine for the synthesis of homocysteine which serves as the immediate precursor to methionine. In combination with the action of the other transsulfuration and reverse transsulfuration reactions catalyzed by cystathionine γ-lyase, cystathionine γ-synthase, and cystathionine β-synthase (shown in Figure 1), this allows for either cysteine or methionine (via homocysteine) production from the intermediate cystathionine [1]. In yeast, secondary reactions of cystathionine β-lyase appear to involve aromatic thiol precursors (e.g., releasing 3-mecaptohexan-1-ol) [2]. Cystathionine β-lyase has been the subject of structural active-site characterization studies [3] and, due to its presence in bacteria and absence in humans, is a target enzyme for the development of novel antimicrobial agents [4]. Cystathionine β-lyase has also been overexpressed in higher plants in an attempt to enhance methionine biosynthesis [5].

*Neurospora crassa* provides a useful model system to dissect the regulation and dynamics of the transsulfuration pathway [6-8]. Early work with *met-2* mutants of *N. crassa* confirmed that they lacked cystathionine β-lyase activity [9]. In *N. crassa,* cystathionine β-lyase has been purified and enzymatic activity assayed under several growth conditions [9,10]. The most significant difference reported [10] was that the level of cystathionine β-lyase activity was reduced in wild-type by one-fourth over the reported baseline when grown with methionine supplemented at the highest level tested (i.e., 5 μmoles/ml) with little change noted at lower levels of supplementation. *N. crassa* microarray expression data collected at different stages of development demonstrate only modest changes in *met-2*^+^ expression during, for example, conidial germination or colony development (e.g., 1.4× higher early in germination versus 15 hr of growth) [11,12]. Limited data is available regarding the regulation of cystathionine β-lyase in other fungi. In *Aspergillus nidulans*, the cystathionine β-lyase gene is encoded by the *metG* locus and the gene has been cloned and characterized [13]. Analysis of the transcription of *metG* demonstrated constitutive expression with no apparent regulation by sulfur source [13]. Cystathionine β-lyase activity in *A. nidulans* was found to be repressed by methionine [14], but constitutively present in another study [15]. For *Saccharomyes cerevisiae*, comparable expression data for growth under different levels of sulfur supplementation is not available for cystathionine β-lyase, which is encoded by the STR3 gene [16]. Based on microarray expression data, STR3 expression does increase by 12-fold under fermentation stress response conditions [17]. In addition, the suggestion has been made that there is a role for Gto1 (omega-class glutathione transferase) in the redox regulation of *S. cerevisiae* cystathionine β-lyase [18].

An important question is whether there is a regulatory connection of the transsulfuration-related genes, including cystathionine β-lyase, to the *N. crassa* sulfur regulatory system which is made up of a set of trans-acting regulatory genes and a set of genes encoding a variety of sulfur-related enzymes and transporters. When *N. crassa* is cultured under sulfur-limiting conditions (i.e., derepressing conditions) then the coordinate expression of this set of sulfur-related genes occurs. The regulated genes include arylsulfatase, choline sulfatase, sulfate permease I and II, among others [6,8]. Recently, cystathionine γ-lyase has been added to the list of genes confirmed to be under control of this key regulatory circuit [19]. CYS3, a bZIP transcriptional activator, is the key regulator and necessary for regulation expression of these sulfur-related genes [6]. Based on binding-site studies, a consensus sequence for CYS3 binding has been determined [20].

We present here the cloning and regulatory analysis of the *N. crassa met-2*^*+*^ cystathionine β*-* lyase gene. This report demonstrates a new connection of cystathionine β-lyase to the CYS3-controlled regulatory network and provides us with important data for the continued dissection of the regulatory dynamics of transsulfuration.

## Results and discussion

### Sequence and characterization of the cystathionine β-lyase gene

**Figure 2:**
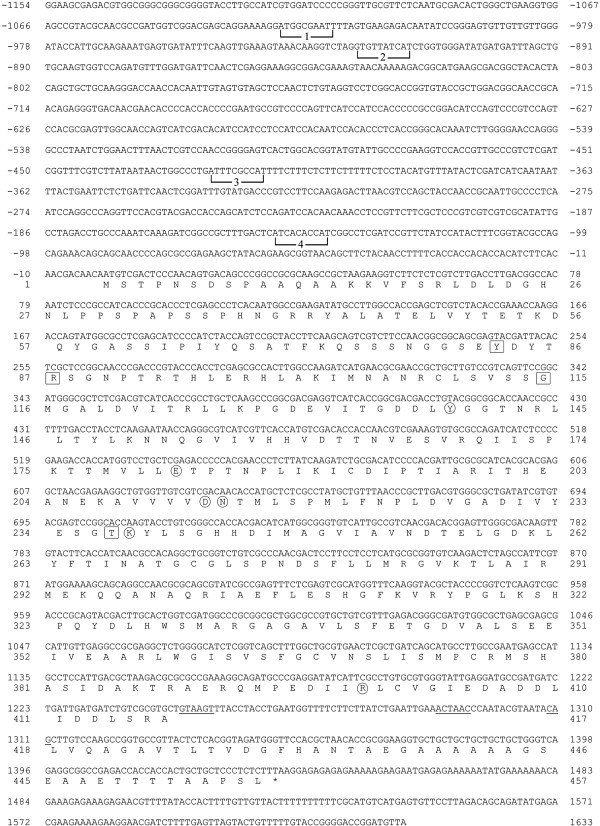
**Nucleotide sequence of the *met-2***^***+***^**gene.** The sequence is shown from 1066 nucleotides upstream of the translation start codon to 187 nucleotides downstream of the stop codon (indicated by an asterisk). The nucleotides are numbered relative to the initiator ATG codon. The predicted polypeptide sequence is given below the nucleotide sequence in single-letter code. The 5′ splice site, the 3′ splice site, and the internal lariat sequence within the intron are underlined. Putative CYS3-binding sites within the *met-2*^*+*^ promoter are bracketed and numbered 1 through 4. Conserved functional residues are highlighted within the predicted polypeptide sequence of 457 amino acids. Residues involved in cofactor binding are boxed; while residues involved in catalysis (including Lys238 involved in the Schiff base linkage) are circled.

The nucleotide sequence of the *met-2*^*+*^ gene along with flanking 5′ and 3′ regions is shown in Figure 2. The *met-*2^+^ gene, which has been mapped to chromosome IVR [21], was cloned by transforming the methionine-requiring *met-*2 mutant (FGSC # 4061) to prototrophy using chromosome IV-specific genomic clones from the Orbach/Sachs *N. crassa* library constructed in pMOCosX [22]. The cosmid vector pMOCosX carries a hygromycin resistance marker for selection [23] which allowed for less background growth following transformation during the screening process. A single clone from the library, designated G8F01 was found to transform *met-2* to prototrophy. A subcloned 4.1 kb fragment capable of transforming the *met-2* mutant was then sequenced for further study. The *met-2*^*+*^ gene was cloned and the sequence submitted as GenBank AF401237 prior to the completion of the *N. crassa* genomic sequence. Subsequently, the locus has been designated NCU07987 in the Broad Institute database [24]. The GenBank AF401237 sequence features as reported here are identical to those reported in NCU07987. The *met-2*^*+*^ gene has a single intron that is 63 nucleotides in length near the 3′ end of the gene as shown by cDNA and genomic sequence data. A putative transcriptional start, with the sequence TCATCACA, is located at −149 upstream of the initiator ATG. Putative binding sites for the CYS3 regulator, identified in experiments as described below, are included in Figure 2. The *met-2*^*+*^ gene encodes a polypeptide of 457 amino acids with important functional residues conserved as compared to other members within this group of pyridoxal-5′-phosphate dependent lyases [3,25,26]. In particular, conserved key residues include Tyr83, Arg87, Gly115, and Thr237 involved in cofactor binding; and Tyr139, Glu182, Asp213, Asn214, Lys238 (the residue forming the Schiff base linkage) and Arg399 involved in catalysis (Figure 2). The *N. crassa* cystathionine β-lyase shows substantial similarity to fungal (e.g., 69% identity to *A. nidulans* AABB03241 and 50% identity to *S. cerevisiae* NP_011331) and to plant (43% identity to *Arabidopsis thaliana* NP_850712) cystathionine β-lyases.

### Analysis of *met-2*^*+*^ gene expression

**Figure 3:**
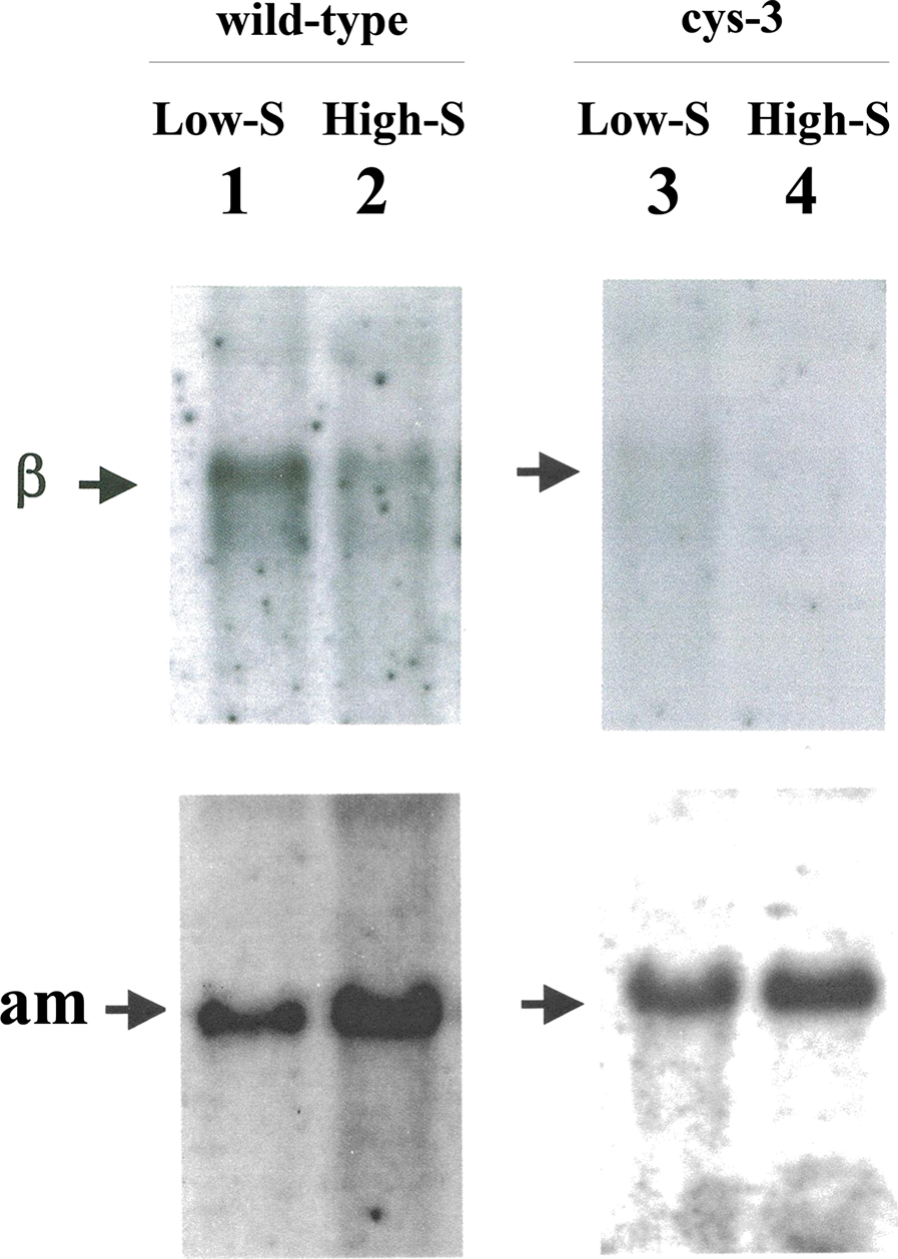
**Analysis of *met-2***^***+***^**expression. (Left panel) Northern hybridization analysis of poly (A)**^**+ **^**mRNA from wild-type *N. crassa *grown under low sulfur (or derepressing) (lane 1) and high sulfur (or repressing conditions) (lane 2).** Northern blots were probed with ^32^P-labeled *met-2*^*+*^ DNA (designated β within figure) and *am*^*+*^ DNA (which served as a constitutively expressed control to allow for sample comparisons). These results are directly comparable to the prior *cys-16*^*+*^ study [19]. (Right panel) Northern analysis of poly (A)^+^ mRNA isolated from the ∆*cys-3* (18–4) strain of *N. crassa* grown under low sulfur (or derepressing) (lane 3) or high sulfur (or repressing) (lane 4) conditions. Blots were probed as above.

*met-2*^*+*^ transcript levels were initially assayed in wild-type *N. crassa* cultured on high and low levels of sulfur (i.e., repressing and derepressing conditions, respectively). Northern blots were prepared from isolated poly (A)^+^ mRNA and probed with the cloned *met-2*^*+*^ gene. Figure 3 shows a 1.7-kb message hybridizing to the *met-2*^*+*^ probe that was detectable under both sulfur repressing and derepressing conditions. The steady-state level of *met-2*^*+*^ transcript did, however, show a substantially higher level under sulfur derepressing conditions (3.1-fold more compared to the sulfur repressing condition based on phosphorimager quantitation of the data). All of the Northern blot experiments conducted used the constitutively expressed *am*^*+*^ gene, which encodes glutamate dehydrogenase, as a control probe for the blots to ensure that the bulk mRNA levels were comparable between samples. Given that in wild-type *N. crassa* the steady-state level of *met-2*^*+*^ transcript was substantially higher under sulfur derepressing conditions, an expression pattern similar to what has been observed for other components of the *N. crassa* sulfur regulatory circuit [6], the results suggest a possible role for CYS3 in the transcriptional control of *met-2*^*+*^. In particular, in relation to transsulfuration, *met-2*^*+*^ expression appears regulated in a similar fashion as with *cys-16*^*+*^ (which encodes cystathionine γ-lyase) [19]. The *met-2*^*+*^ levels observed in Figure 3 are directly comparable to those in our prior study with the *cys-16*^*+*^ locus [19]. The expression data suggests, at least in *N. crassa*, that the available pool of cystathionine is accessed by derepressing both cystathionine β-lyase (leading to homocysteine and subsequently methionine) and by cystathionine γ-lyase (leading to cysteine) when the organism is stressed by low sulfur availability (pathway shown in Figure 1). This is in marked contrast to the other similarly characterized fungal example, *A. nidulans*, where constitutive expression of cystathionine β-lyase has been shown [13].

In order to test whether *met-2*^*+*^ is subject to regulation by CYS3, Northern blot analysis was carried out using poly(A)^+^ mRNA isolated from a *cys-3* mutant deletion mutant (Δ*cys-3*) and probed with the *met-2*^*+*^ gene. For this case, the *met-2*^*+*^ transcript was detectable only at a very low level under either low- or high-sulfur culture conditions. In fact, the levels observed in *Δcys-3* were slightly lower than in wild-type under repressing conditions (based on phosphorimager quantification). The reduction in *met-2*^*+*^ transcript level in the Δ*cys-3* background is typical of genes that have been demonstrated to be part of the *N. crassa* sulfur regulatory system in previous studies [6,8].

### Gel mobility shift analysis of the *met-2*^*+*^ promoter

**Figure 4:**
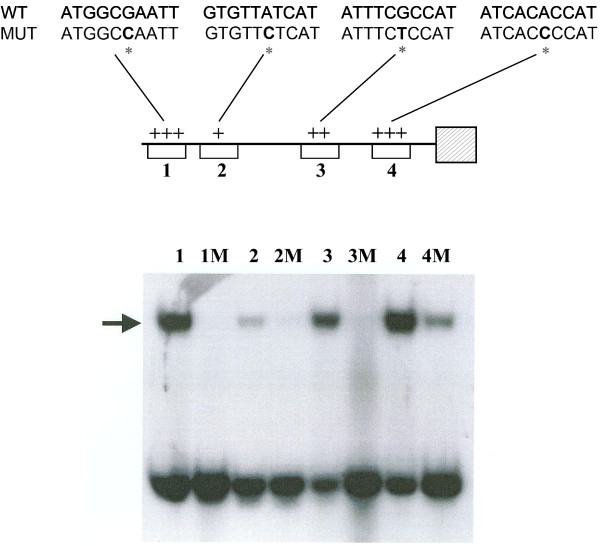
**Gel mobility-shift analysis of CYS3 binding sites within the *met-2 ***^**+ **^**promoter. Lanes: 1, CYS3-binding site 1 (nt −1017 to −1026); 1 M, mutated CYS3-binding site 1; 2, CYS3-binding site 2 (nt −916 to −925); 2 M, mutated CYS3-binding site 2; 3, CYS3-binding site 3 (nt −413 to −422); 3 M, mutated CYS3-binding site 3; 4, CYS3-binding site 4 (nt −138 to −147); 4 M, mutated CYS3-binding site 4.** Each CYS3 binding site was mutated with a single point mutation to determine the specificity of CYS binding.. The arrow indicates DNA fragments exhibiting reduced electrophoretic mobility due to protein-DNA interactions. The schematic of the *met-2*^*+*^ promoter (top) depicts the affinity of CYS3 for each binding site (+, weakest affinity; +++ strongest affinity) and includes the corresponding binding site nucleotide sequences for both wild type (WT) and mutated (MUT).

Based on *in vitro* binding and other studies the consensus binding site for CYS3 has been determined to be 5′ ATG GCGC CAT 3′ [20] . In order to analyze the *met-2*^*+*^ gene for the presence of CYS3 binding sites, the promoter region was subdivided into overlapping 100- to 200-bp segments by restriction endonuclease digestion and examined for gel mobility shifts with CYS3 extract (data not shown). The fragments demonstrating mobility shifts were progressively narrowed down to 24 bp segments that were still able to bind CYS3. Figure 4 shows that among the group of defined segments, CYS3 binding was strongest to site 4 (at −138 to −147) which had a sequence of 5′ ATC ACAC CAT 3′ which corresponded to the closest match to the CYS3 consensus binding site found in the *met-2*^*+*^ promoter. Previous studies have shown that the core of the consensus binding site commonly contains RYRY variations as in 5′ ATG RYRY CAT 3′) [8,20]. The weakest binding was observed at site 2 (−916 to −925) which had the greatest divergence from the consensus binding site (i.e., 5′ GTG TTAT CAT 3′). To confirm the specificity of CYS3 binding, the sixth base position within the 10 bp core sequence was mutated (from purine to pyrimidine), and the effect on gel mobility shifts determined. Figure 4 shows that the mutated segments showed no or reduced binding of CYS3and, therefore, demonstrate the specificity of the binding site assay.

## Conclusions

The expression of the *N. crassa met-2*^*+*^ gene, which encodes cystathionine β-lyase, was found to increase in level under culture conditions of sulfur limitation. Further, the reduced *in vivo* expression of *met-2*^*+*^ seen in a Δ*cys-3* background along with the *in vitro* detection of CYS3 binding sites within the *met-2*^*+*^ promoter supports a role for CYS3-mediated regulation of this gene. The data add to the continuing analysis of the regulatory dynamics of transsulfuration in this model system.

## Methods

### Strains, plasmids and culture conditions

The *met-2* mutant strain FGSC#4061 and Orbach/Sachs *N. crassa* genomic library [22] that was constructed with the cosmid pMOCosX [23] were obtained from the Fungal Genetics Stock Center (University of Missouri-Kansas City). The ∆*cys-3* (18–4) deletion strain was constructed as described previously [27]. The strain 74OR23-1a was used as wild-type for these experiments. Vogel minimal medium [28], with supplements as required, was used. Sulfur repression and derepression experiments were carried out as described previously by culturing mycelia at 25°C on Vogel-minus-sulfur medium plus high sulfur (5.0 mM methionine) and low-sulfur medium (0.25 mM methionine) medium, respectively [29].

### Gene cloning and sequencing

The *N. crassa* cosmid library was subdivided into chromosome IV specific clones which were then used in individual transformations of *met-*2 with selection for methionine prototrophy and hygromycin resistance. A single clone designated G8F01 was found that was capable of transforming *met-*2. Subsequent *met-*2 transformation tests identified a 4.1 kb fragment which contained the *met-2*^*+*^ gene. Following subcloning into pSPORT, the segment was subjected to automated sequencing (Cleveland Genomics; Cleveland, OH) by primer walking. The genomic sequence was submitted to GenBank as AF401237. The *met-2*^*+*^ gene has been assigned the locus designation NCU07987 in the Broad Institute *Neurospora crassa* database [24].

### Northern analysis

Total RNA was isolated by the phenol extraction procedure described previously [29]. Following sodium acetate washes, poly(A)^+^ mRNA was isolated by oligo(dT) cellulose chromatography. Oligolabeling of DNA fragments was used to prepare ^32^P-labeled probes and used to probe Northern blots prepared as outlined elsewhere [29]. Quantitation of Northern blots was accomplished by using a Molecular Dynamics Phosphorimager.

### Gel mobility shifts

Initially, the *met-2*^*+*^ promoter was subjected to restriction endonuclease digestion to generate a series of overlapping fragments tested in gel mobility shift assays. Subsequent identification of fragments that bound CYS3 allowed for the synthesis of short segments (24 bp) that retained binding activity. The 24 bp oligonucleotides representing the binding sites to be further tested were synthesized with an Applied Biosystems 391EP synthesizer, labeled using [ϒ-^32^P]ATP, annealed and gel purified as described previously [20,30]. Four oligonucleotides (and complementary strands) representing putative CYS3 binding sites on the *met-2*^*+*^ promoter were analyzed: Site 1 [ 5′ GAAAAGGATGGCGAATTTTAGTGA 3′], Site 2 [ 5′ GGTCTAGGTGTTATCATCTGGTGG 3′], Site 3 [5′GGCCCTGATTTCGCCATTTTCTTT 3′], and Site 4 [5′ TTGACTCATCACACCATCGGCCTC 3′]. Mutated CYS3 binding sites had a purine to pyrimidine substitution at the sixth position of the 10 bp core of the CYS3 consensus binding site: Site 1 M [ 5′ GAAAAGGATGGC**C**AATTTTAGTGA 3′], Site 2 M [5′ GGTCTAGGTGTT**C**TCATCTGGTGG 3′], Site 3 M [5′ GGCCCTGATTTC**T**CCATTTTCTTT 3′], and Site 4 M [5′ TTGACTCATCAC**C**CCATCGGCCTC 3′]. *E. coli* produced CYS3 protein [20] was used for gel shift assays as we have described [20,30]. A Molecular Dynamics Phosphorimager was used to quantify the gel shift binding assays.

### Availability of supporting data

The sequence data supporting the results of this article is available in the GenBank respository [AF401237, http://www.ncbi.nlm.nih.gov/nuccore/AF401237].

## Competing interests

The authors declare that they have no competing interests.

## Authors’ contributions

BR carried out the majority of experiments. JP and BR designed the experiments and prepared the manuscript. Both authors read and approved the final manuscript.
